# MODIS Derived Sea Surface Salinity, Temperature, and Chlorophyll-a Data for Potential Fish Zone Mapping: West Red Sea Coastal Areas, Saudi Arabia

**DOI:** 10.3390/s19092069

**Published:** 2019-05-03

**Authors:** Saleh T. Daqamseh, A’kif Al-Fugara, Biswajeet Pradhan, Anas Al-Oraiqat, Maan Habib

**Affiliations:** 1 Department of Human Sciences, Geography, Art faculty, Taibah University, P.O. Box 2898, Medina 41477, Saudi Arabia; sdaqamseh@taibahu.edu.sa; 2 Department of Surveying Engineering, Faculty of Engineering, Al al-Bayt University, Mafraq 25113, Jordan; 3 Centre for Advanced Modelling and Geospatial Information Systems (CAMGIS), School of Information, Systems & Modelling, Faculty of Engineering and IT, University of Technology Sydney, Sydney, NSW 2007, Australia; 4 Department of Energy and Mineral Resources Engineering, Choongmu-gwan, Sejong University, Seoul 05006, Korea; 5 Department of Computer Sciences and Information, Community College, Taibah University, Medina 41477, Saudi Arabia; anas_oraiqat@hotmail.com; 6 Department of Surveying and Geomatics Engineering, Al-Balqa Applied University, Al Salt 19117, Jordan; maan.habib@gmail.com

**Keywords:** remote sensing, salinity, temperature, chlorophyll-a concentration, MODIS, potential fish zone

## Abstract

In this study, a multi-linear regression model for potential fishing zone (PFZ) mapping along the Saudi Arabian Red Sea coasts of Yanbu’ al Bahr and Jeddah was developed, using Moderate Resolution Imaging Spectroradiometer (MODIS) satellite data derived parameters, such as sea surface salinity (SSS), sea surface temperature (SST), and chlorophyll-a (Chl-a). MODIS data was also used to validate the model. The model expanded on previous models by taking seasonal variances in PFZs into account, examining the impact of the summer, winter, monsoon, and inter-monsoon season on the selected oceanographic parameters in order to gain a deeper understanding of fish aggregation patterns. MODIS images were used to effectively extract SSS, SST, and Chl-a data for PFZ mapping. MODIS data were then used to perform multiple linear regression analysis in order to generate SSS, SST, and Chl-a estimates, with the estimates validated against in-situ data obtained from field visits completed at the time of the satellite passes. The proposed model demonstrates high potential for use in the Red Sea region, with a high level of congruence found between mapped PFZ areas and fish catch data (R^2^ = 0.91). Based on the results of this research, it is suggested that the proposed PFZ model is used to support fisheries in determining high potential fishing zones, allowing large areas of the Red Sea to be utilized over a short period. The proposed PFZ model can contribute significantly to the understanding of seasonal fishing activity and support the efficient, effective, and responsible use of resources within the fishing industry.

## 1. Introduction

In human history, large coastal populations around the world are reliant on fisheries, with fish having provided humans with essential sustenance for thousands of years [1]. Consequently, fish and seafood play an important role in food security for many individuals, families, and communities. However, researchers, such as [2], reported that developing countries have published little information about the availability of fish and seafood—or its impact on food security and local incomes—due to the absence of data on fish and fisheries. This is of significant concern given that demand for fish is growing every year in line with population growth, with fisheries needing to produce higher numbers of fish to feed local populations. The Food and Agriculture Organization of the United Nations [3] notes that the Northern Red Sea represents a key fishing region due to its rare and bountiful seagrass beds, mangroves, coral reefs, and overall marine environment. The Red Sea is one of the world’s most important water bodies, connecting the Mediterranean Sea through the northern Suez Canal and to the Indian Ocean through the southern Bab el-Mandeb Strait. Further, [3,4] noted that fisheries play a crucial role in supporting the economies of these bordering countries, with fisheries in this region often sharing stocks from the Red Sea.

Recent progress in the development of satellite sensors, image-processing techniques, sea data collection techniques, and remote sensing modelling have together provided a powerful tool for ocean parameter analysis and monitoring [5,6]. Researchers, such as [7], also reported the use of data obtained from satellite technology in mapping potential fishing zones (PFZs), as well as ocean parameter analysis and other marine activities achieved through the use of L-band microwave technology [8]. A number of researchers, including [9,10,11,12], have tested the ability to map PFZs using advanced very high-resolution radiometer (AVHRR) technology. Despite this progress, very few researchers have taken into account the ways in which PFZ maps are impacted by sea temperature and salinity, with most researchers also considering the shoreline as being a strong PFZ candidate due to the high reflectivity of Chlorophyll-a (Ch1-a) in these areas. Consequently, the accuracy of PFZ mapping achieved by such studies has been called into question.

Moderate resolution imaging spectro-radiometer (MODIS) algorithms have been found to be of greater value than alternative remotely sensed data in sea data extraction, according to previous modelling studies conducted around the world on a daily basis across seven spectral bands. For instance, [13,14,15] developed different retrieval algorithms for sea surface temperature (SST) based on MODIS data. Further, [16,17,18,19] have developed linear and nonlinear algorithms to measure sea surface salinity (SSS) using MODIS data; and [20,21,22,23,24,25] used MODIS data to measure Chl-a.

Other researchers have presented algorithms and tested the validity of PFZ mapping using in-situ data. However, the chosen sites were small and seasonality was not taken into account, leading to models with low PFZ mapping accuracy and ocean parameter extraction [26,27,28,29,30]. The accuracy of PFZ models in these studies was also low due to the dependence on SST or combined SST/Chl-a measures. Since the selected areas featured shallow waters, the shorelines were characterized by highly-reflective chlorophyll related to the comparative concentrations of optically-active constituents, such as phytoplankton and suspended particulate matter, resulting in inaccurate PFZ models that considered shorelines to have a high potential for fishing activity.

One of the ways in which global warming and climate change can be measured is to examine population levels of cold-water fish (salmonids) and aquatic invertebrates since they cannot survive significant changes in the temperature of their immediate local environment. The International Union for Conservation of Nature [31] has reported that over 4161 species are at risk due to climate change and environmental issues, such as changes to natural habitats (impacting 33% of species), high or low temperatures (29% of species), and drought (28% of species). Given these findings, there is an evident need for greater consideration of SST and SSS in PFZ mapping research. This reflects the primary aim of the current study, which was to achieve accurate PFZ mapping in the selected area of the Red Sea. Therefore, in this study, fish density and catch-per-unit-effort were measured along with both the biological and physical attributes of SSS, SST, and Chl-a.

## 2. Materials and Methods

### 2.1. Study Area

**Figure 1:**
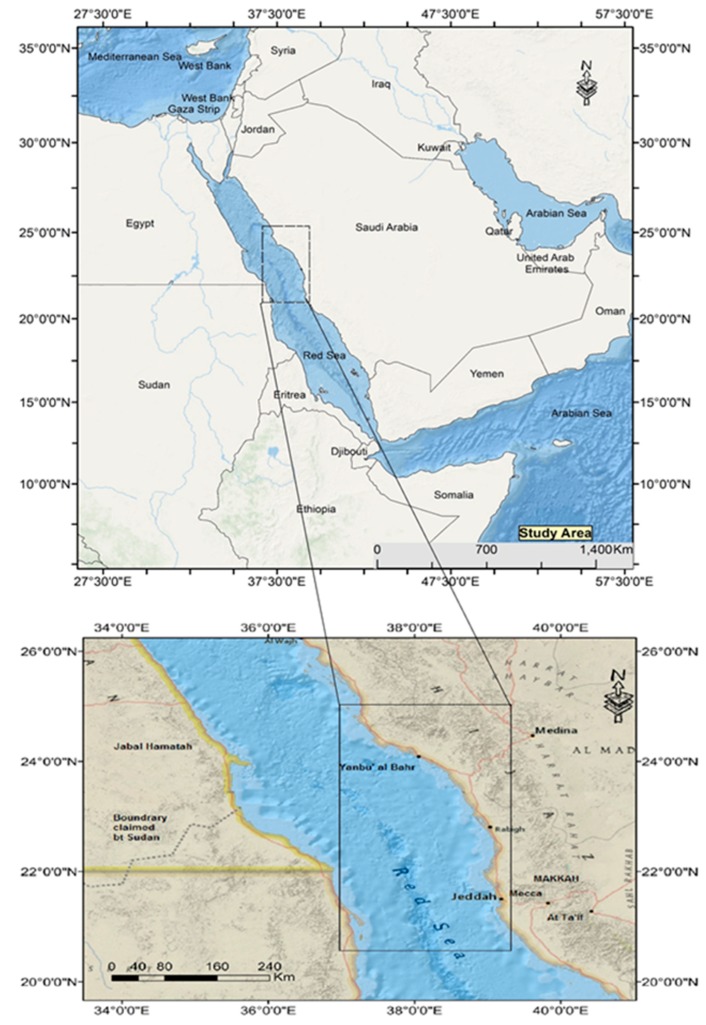
Study area map (Red Sea, Saudi Arabia).

The location of the sampling locations is illustrated in Figure 1 (black box). The study site is located in Western Saudi Arabia (latitude 21° 15′00″ to 25° 00′30″ N; longitude 37° 45′30″ to 41° 00′30″ E), spanning along Jeddah and Yanbu’ al Bahr cities. The site is bordered by Saudi Arabia to the east and Sudan to the west. The Red Sea itself is about 2000 km long and 300 km across at its widest part, with a surface area of 450,000 km^2^, and is located between the Asian and African coastlines. It is situated at a latitude of 12° N to 30° N and longitude of 32° E to 44° E. With an irregular topography, the deepest area of the Red Sea features waters of approximately a 3 km deep, providing a prime environment for marine life. The selected study site is well-representative of the bathymetric features of the coastal zones of the Red Sea, including coral reefs and shoals.

### 2.2. Data

#### 2.2.1. In-Situ Measurements

**Figure 2:**
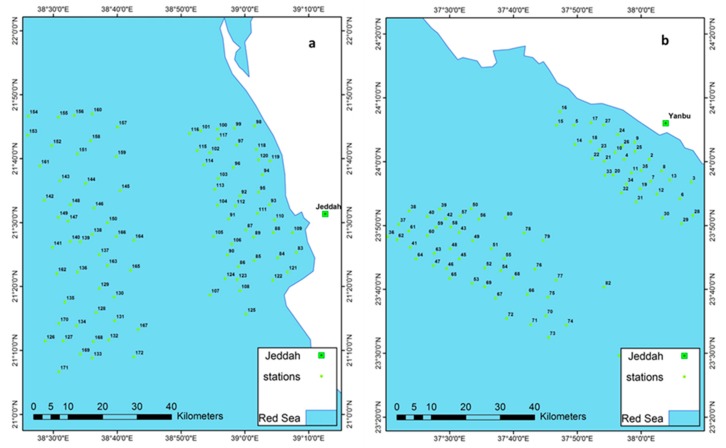
Locations of sampling stations during two cruises, a. Jeddah cruises (June, July, August, and November 2015), and b. Yanbu’ al Bahr cruises (coastline in February 2014).

Measurements were taken at the selected sites in the Red Sea over a period of 10 months (June 2014–March 2015), with two trips made via sea cruises along the Jeddah coast during the same time as the satellite passes. The field campaign was carried out over large-scale sampling areas, exceeding 56 km^2^. During each trip, the samples were taken from the Jeddah coastline over seven clear days in June, July, August, and November 2015 with a total of 47 sampling sites at the open sea area in Jeddah with an interval of about 500 m between two samples, and 43 sampling sites for the inshore area with an interval of about 250 m between two samples. Also, samples were taken at vertical distribution at an average depth of 1 to 2 m (near shore), and at an average depth of 20 to 100 m (open sea). Additionally, in-situ measurements were also taken along the coast of the Yanbu’ al Bahr coastline in February 2014, with a total of 47 sampling sites at the open sea area in Yanbu’ with an interval of about 500 m between samples’ locations, and 37 sampling sites for the inshore area with an interval of about 250 m between samples’ locations. Figure 2 illustrates the location map of each cruise station. In addition, Hydro-Lab MS was utilized for the measurement of SSS and SST, and Hydro-lab Datasonde Multi-Parameter Water Quality Sonde (HACH, USA) was used for the collection of Chl-a. Continuous fluorescence vertical profiles were collected at each station. The fluorescence sensor was calibrated in the laboratory prior to each cruise using two-point calibration, including a blank (de-ionized water with zero Chl-a concentration (0 µg L^−1^), and another point with a non-zero value, which was selected to be at the middle range of the Red Sea Chl-a concentration values (0.2 µg L^−1^). The non-zero Chl-a concentration was a commercial sample of spinach standard obtained from Sigma-Aldrich, (part code C5753). Each Chl-a vial contained 1 mg of pure chlorophyll, in which each vial’s content was dissolved in 50 mL of high-performance liquid chromatography (HPLC) grade (90% acetone) to give 20 mg/L stock solutions, which were further diluted when needed in 90% acetone to give solutions of 0.2 µg L^−1^. Unlike the Chl-a Hydrolab Datasonde probe, conductivity and salinity sensors were calibrated before and after each day of data collection against standard solutions, while the temperature did not require any calibration as it was factory calibrated. The parameters recorded were: Conductivity in mS/cm, SSS in Ppt, SST in degrees Celsius, and Chlorophyll-a in ug/L. The instrument was attached to the data cable and secured with a rope before being lowered into the water column for measurements (the vertical distribution of the temperature of the nearshore ranged from 31 °C to 36 °C, while the temperature range of the vertical distribution at open sea waters was between 12 °C and 37.96 °C. Vertical distribution of salinity in nearshore waters ranged from 39 to 40.96. The vertical distribution of salinity was seen to be in an increasing trend in deep waters ranging between 38 to 43.8, with 1 difference of salinity between the minimum salinity of shallow waters and deep waters. At least two spectral measurements were taken during each sampling activity in order to match the spatio-temporal MODIS reflectance. An average reflectance measurement was used to feed into the regression equation. The corresponding image reflectance of a 3 × 3 pixel box was centered on an in situ data point, which was calculated at the same time when the MODIS overpassed the sample locations. Simultaneously, Hydrolab Datasonde instruments were set up to record water quality measurements in the real-time mode [6].

#### 2.2.2. MODIS Satellite Data

The satellite data for the period of February 2014 to March 2015 were downloaded from the MODIS website (http://modis.gsfc.nasa.gov/data/). At the same time, in-site measurements were made. A sequence of MODIS 500-m and 1-km Terra and Aqua images were acquired, pre-processed, and analyzed to construct time series of SST, SSS, and Chl-a data using the appropriate models described in Section 3 in detail. The MODIS images used in this study were corrected geometrically and the orbit overlap and swath distortion (the bow-tie effect) were fixed using the “Georeference MODIS” function in ENVI software 4.2 version. The correction was done with the intention of comparing the image data with water quality monitoring stations. The coastline vector data were overlaid onto the MODIS images and visually examined so that the shift between both images and the vector coastline did not exceed 0.5 pixels. Then, the images were normalized with the corresponding spectral range using the empirical line calibration method for the purpose of getting rid of illumination and atmospheric effects [12,13]. Pre-processing of the MODIS data was carried out by masking the land and cloud to avoid interference of the reflectance of the cloud and land with reflectance of the ocean parameters of the model (SSS, SST, and Chl-a). The data ranged from February 2014 to March 2015, in line with the sampling dates of this research. In order to generate time series of SST, SSS, and Chl-a data through the use of the relevant models, MODIS 500 m and 1 km Terra and Aqua images were obtained. Land and cloud masking was performed for the purpose of pre-processing the MODIS data to ensuring that the accuracy of SST, SSS, and Chl-a reflectance would not be hindered by cloud and land reflectance.

## 3. MODIS Satellite Data Extraction: SSS, SST, and Chl-a

Ocean parameter monitoring and analysis has been developed significantly over recent years as a result of the development of geographic information system (GIS) modelling, satellite sensors, sea data collection approaches, and image processing methods [5,6,25,26]. The techniques adopted in the present study are outlined in line with these developments.

### 3.1. Sea Surface Salinity (SSS)

The initial model adopted in this study was the MODIS-Aqua SSS algorithm [26], which was developed for a study focused on the east coast of Malaysia and the South China Sea. The main assumption of the model is that the MODIS image radiance I within multi-channels has a linear relationship with the measured SSS [6,26]. To retrieve the SSS from the observed ocean salinity brightness temperatures (TBs), a multiple linear regression was used to model the relationship between both TBs and in situ measurements. The model used in-situ SSS measurements and was built upon multiple linear regression analysis, using 1 to 7 MODIS bands: B1_(620–670)_, B2_(841–876)_, B3_(459–479)_, B4_(545–565)_, B5_(1230–1250)_, B_(61628–1652)_, and B7_(2105–2155)_, as there was no single band that had a significant correlation with salinity using these bands. Therefore, multiple linear regressions were employed to estimate the relationship between SSS extracted from MODIS imageries’ thermal infrared bands of a 250- to 500-m resolution and used for SSS observation of MODIS bands: B8_(405–420)_, B9_(438–448)_, B10_(483–493)_, B11_(526–536)_, B12_(546–556)_, B13_(662–672)_, B14_(673–683)_, B15_(743–753)_, and B16_(862–877)_, with in situ data. The SSS multiple linear regression model developed by this study was subjected to validation with in situ measurements. To this end, the regression modeling approach was employed. Upon implementing the model to collect SSS measurements from the selected Red Sea sites, some issues were encountered with respect to the accuracy because of the unique nature of the climate and environment in each body of water. We also encountered issues with MODIS band 1–7 SSS extraction given that aerosols, clouds, and land are typically associated with these bands, whilst ocean color and biogeochemical measurements are typically associated with bands 8 to 16. Equation (1), below, presents the regression equation for the relationship between independent (explanatory) variable B (band wavelength B8, B9, …, B16) and the dependent (outcome) variable (SSS). Multiple linear regression was used to model the relationship between these variables based on the collected data:

(1) $$\left[\begin{array}{c} {\mathrm{SSS}}_{1}\\ {\mathrm{SSS}}_{2}\\ \vdots \\ {\mathrm{SSS}}_{n} \end{array}\right]=\left[\begin{array}{ccc} \begin{array}{c} \begin{array}{ccc} 1 & B_{81} & B_{82}\\ 1 & B_{91} & B_{92}\\ \vdots & \vdots & \vdots \end{array}\\ \begin{array}{ccc} 1 & B_{n1} & B_{n2} \end{array} \end{array} & \begin{array}{c} \ldots \\ \ldots \\ \ldots \end{array} & \begin{array}{c} B_{8P}\\ B_{9P}\\ \vdots \\ B_{\mathrm{nP}} \end{array} \end{array}\right]\cdot \left[\begin{array}{c} \alpha_{0}\\ \alpha_{1}\\ \vdots \\ \alpha_{P} \end{array}\right]+\left[\begin{array}{c} e_{1}\\ e_{2}\\ \vdots \\ e_{n} \end{array}\right],$$

for 1, 2, …, n and J = 1, 2, …, p, where SSS_i_ is the observed sea surface salinity value, and ${S\hat{S}S}_{n}$ is the predicted sea surface salinity value; B_nP_ is the P^th^ predictor value for the P^th^ value of band n; α_0_ is the regression constant; α_P_ is the coefficient of the P^th^ predictor; P is the total number of predictors; and e_n_ is the error term, calculated using Equation (2):

(2) $$e_{i}=\sum_{i=1}^{n}{\left({\mathrm{SSS}}_{i}-{S\hat{S}S}_{i}\right)}^{2},$$

(3) $$\begin{array}{c} {\mathrm{SSS}}_{psu}=\left(14.256-240.163\mathrm{Band}8\right)-\left(72.53\times \mathrm{Band}9\right)+\left(124.700\mathrm{Band}10\right)+\\ \left(191.266\times \mathrm{Band}11\right)+\left(36.044\times \mathrm{Band}12\right)-\left(11.117\times \mathrm{Band}13\right)-(39.789\times \mathrm{Band}14), \end{array}$$

where SSS_*psu*_ is the sea surface salinty measured in $\frac{g}{\mathrm{Kg}}$ (practical salinity unit).

### 3.2. Sea Surface Temperature (SST)

In order to ensure suitability for the in-situ samples extracted from the selected Red Sea study sites, four different models were used to obtain SST data from the MODIS satellite data. These models are based on the inherent optical properties and reflectance ranges of the specific study site [13,14,15,26]. Therefore, in this study, the most common models were used to examine the suitability of a number of the existing algorithms for the Red Sea environmental setting, and to identify the one(s) which can provide the most accurate estimates of SSS, SST, and Chll-a. These models were the Brown-Mint [31], Virgini [32], Walton [33], and Wong models [34]. Based on the work of [35], Equation (4) was used to calculate the SST value by using the ATBD-MOD25 Walton algorithm-based ERDAS window method applied to 24 MODIS scenes:

(4) $$\mathrm{SST}=c_{1}+c_{2}\times T_{11}+c_{3}\left(T_{11}-T_{12}\right)\times T_{sfc}+c_{4}\times \left(\sec\left(\theta \right)-1\right)\times \left(T_{11}-T_{12}\right).$$

This equation is based on the non-linear algorithm for SST estimation, where *T_n_* is the brightness temperature (measured at the n µm wavelength in the channels); Tsfc is a climatological estimate of the site’s SST; θ is the satellite zenith angle; and *c*_1_, *c*_2_, *c*_3_, and *c*_4_ are coefficients for the MODIS Band 31 and 32 SST retrieval algorithm [35], given as follows:

*c*_1_ = 1.228552 1.692521,
*c*_2_ = 0.9576555 0.9558419,
*c*_3_ = 0.1182196 0.0873754,
*c*_4_ = 1.774631 1.199584.

### 3.3. Ch1-a

The Chl-a algorithm adopted for MODIS-Aqua in this study is based on ATBD MOD19 [20]. As per the work of [36], it uses an empirical Chl-a algorithm (Chl-a OC3 O’Reilly) that connects the log-transformed remote sensing reflectance ratio, X, to the chlorophyll concentration, Ca. As illustrated in Equations (5) and (6), the OC3 algorithm uses a three-band blue-green reflectance ratio:

(5) $$R=\log10\left[\frac{R_{rs443}>R_{rs488}}{R_{rs551}}\right],$$

(6) $$Ca=10^{0.28-2.753R+1.457\;R^{2}+0.659R^{4}},$$

where *Ca*: Chlorophyll-a concentration (milligram per cubic meter, mg·m^−3^); Rrs: Remote sensing reflectance; and R: Blue-green band ration (dimensionless).

As [37] noted, the Red Sea is typically characterized by chlorophyll concentrations of below 0.25 mg·m^−3^. Therefore, the algorithm developed by [38] for low chlorophyll samples was also adopted in this study, since it is known to be superior to the OC3 algorithm for this type of sample. Dierssen and Kayla’s algorithm is a band-difference technique that operates on a color index (*CI*) representing the difference between Rrs in the visible spectrum’s green region and a linearly formed reference between Rrs in its red and blue regions. Equation (7) illustrates the definition of the *CI* [39]:

(7) $$CI=R_{rs}\left(555\right)-0.5\left[R_{rs}\left(443\right)+R_{rs}\left(670\right)\right].$$

Regression analysis was performed simultaneously with respect to the satellite passing. The regression analysis was carried out on the relationship between seasonal in-situ SST, chlorophyll data, and Chl-a estimates (generated by the adopted algorithm using MODIS data) from the Red Sea samples in order to validate the SST and Chl-a models.

### 3.4. Improving PFZ Mapping with SSS, SST, and Chl-a

Regression analysis was used to measure the statistical impact of the three selected oceanographic parameters for determining PFZs in the chosen Red Sea sites (Table 1). Regression analysis typically involves an estimation of the conditional expectation of the dependent variable based on the independent variables (*X*_1_, *X*_2_, …, *X_n_*). This means that regression analysis provides an average value for the dependent variable when independent variables remain stable [40]. It was anticipated that the chosen variables would have a linear relationship, with the following equation presented for n cases:

(8) $$\mathrm{PFZ}=\alpha_{0}+\alpha_{1}X_{1i}+\alpha_{2}X_{2i}+\alpha_{3}X_{3i}+\alpha_{4}X_{4i}+\cdots +\varepsilon_{i}\;1\leq i\leq n,$$

where 1 ≤ *i* ≤ *n* is the cases in the sample size (*n*); α_0_, α_1_, α_2_, α_3_, …, and α_*n*_ are the unknown regression equation coefficients; PFZ is a potential fish zone; T is water temperature (in °C); C is Chl-a concentration (µg/L); S is water salinity (); and ε is the residual error present in the estimate generated by the model estimate. Equation (8) can be presented in matrix form as:

(9) $$\left[\begin{array}{c} {\mathrm{PFZ}}_{1}\\ {\mathrm{PFZ}}_{2}\\ \vdots \\ {\mathrm{PFZ}}_{n} \end{array}\right]=\alpha_{0}+\alpha_{1}\left[\begin{array}{c} T_{1}\\ T_{2}\\ \vdots \\ T_{n} \end{array}\right]+\alpha_{3}\left[\begin{array}{c} C_{1}\\ C_{2}\\ \vdots \\ C_{n} \end{array}\right]+\alpha_{4}\left[\begin{array}{c} S_{1}\\ S_{2}\\ \vdots \\ S_{n} \end{array}\right]+\left[\begin{array}{c} \varepsilon_{1}\\ \varepsilon_{2}\\ \vdots \\ \varepsilon_{n} \end{array}\right].$$

This matrix can be derived as follows.

Consequently, Equations (10) and (11) were used to arrive at the matrix:

(10) $$\left[\begin{array}{c} {\mathrm{PFZ}}_{1}\\ {\mathrm{PFZ}}_{2}\\ \vdots \\ {\mathrm{PFZ}}_{n} \end{array}\right]=\left[\begin{array}{ccccc} \alpha_{0} & \alpha_{1} & \alpha_{2} & \alpha_{3} & \alpha_{4} \end{array}\right]\left[\begin{array}{cccc} 1 & 1 & \cdots & 1\\ T_{1} & T_{2} & \cdots & T_{n}\\ C_{1} & C_{2} & \cdots & C_{n}\\ S_{1} & S_{2} & \cdots & S_{n} \end{array}\right]+\left[\begin{array}{c} \varepsilon_{1}\\ \varepsilon_{2}\\ \vdots \\ \varepsilon_{n} \end{array}\right],$$

(11) $$\left[\begin{array}{c} {\mathrm{PFZ}}_{1}\\ {\mathrm{PFZ}}_{2}\\ \vdots \\ {\mathrm{PFZ}}_{n} \end{array}\right]=\left[\begin{array}{c} \alpha_{0}+\alpha_{1}T_{1}+\alpha_{3}C_{1}+\alpha_{4}S_{1}\\ \alpha_{0}+\alpha_{1}T_{2}+\alpha_{3}C_{2}+\alpha_{4}S_{2}\\ \vdots \\ \alpha_{0}+\alpha_{1}T_{n}+\alpha_{3}C_{n}+\alpha_{4}S_{n} \end{array}\right]+\left[\begin{array}{c} \varepsilon_{1}\\ \varepsilon_{2}\\ \vdots \\ \varepsilon_{n} \end{array}\right].$$

The high SST and Chl-a coefficient values in the final regression model (Equation (13)) are indicative of good PFZ locations based on the positive coefficients for the parameters produced via regression analysis. Conversely, low SSS values are thought to be associated with a poor PFZ location based on the negative coefficient for this parameter. As mentioned before, these models are based on the inherent optical properties and reflectance ranges of the specific study site, therefore the calibrated model will forecast different predictive PFZ mapping values at different site locations.

The differences between the PFZ values predicted by the model adopted in this paper (Red Sea) and the earlier model (South China Sea) are illustrated in Equation (12), below:

(12) $$\left[\begin{array}{c} \begin{array}{c} {\mathrm{PFZ}}_{1}\\ {\mathrm{PFZ}}_{2} \end{array}\\ \vdots \\ {\mathrm{PFZ}}_{n} \end{array}\right]=\left[\begin{array}{c} 8302.01-425.58T_{1}+16.367C_{1}+59.458S_{1}\\ 8302.01-425.58T_{2}+16.367C_{2}+59.458S_{2}\\ \vdots \\ 8302.01-425.58T_{n}+16.367C_{n}+59.458S_{n} \end{array}\right]+\left[\begin{array}{c} \begin{array}{c} \varepsilon_{1}\\ \varepsilon_{2} \end{array}\\ \vdots \\ \varepsilon_{n} \end{array}\right].$$

As illustrated in Equation (13), the results indicate that PFZ prediction is most significantly impacted by the SST value; salinity is one of the most extensively studied. In fact, it is a determining factor for the growth of fishes, according to the modified PFZ model, which introduces a new predictive PFZ mapping value:

(13) PFZ=8302.01−(425.58)SST+(16.367)Ch1-a+(59.458)SSS+ε.

## 4. Results and Discussion

### 4.1. SSS: In-Situ versus MODIS

**Figure 3:**
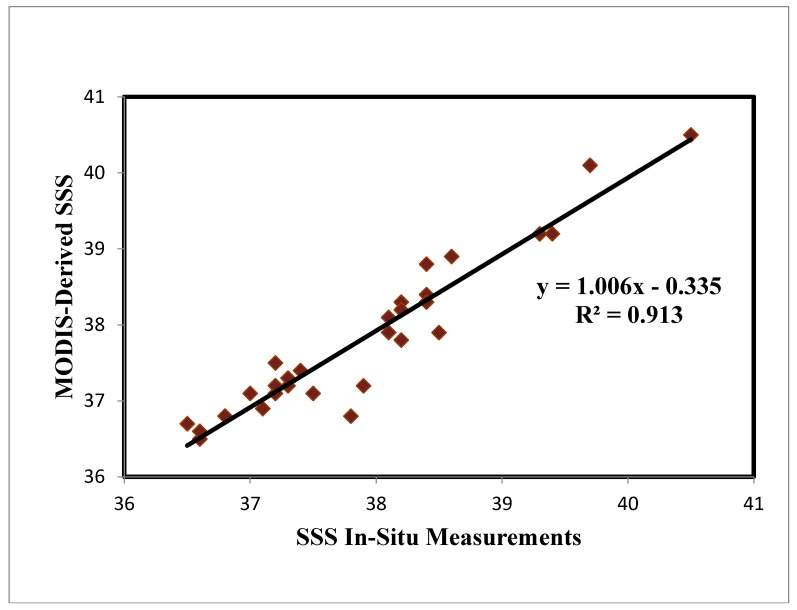
In-situ vs. MODIS-based SSS estimates.

**Figure 4:**
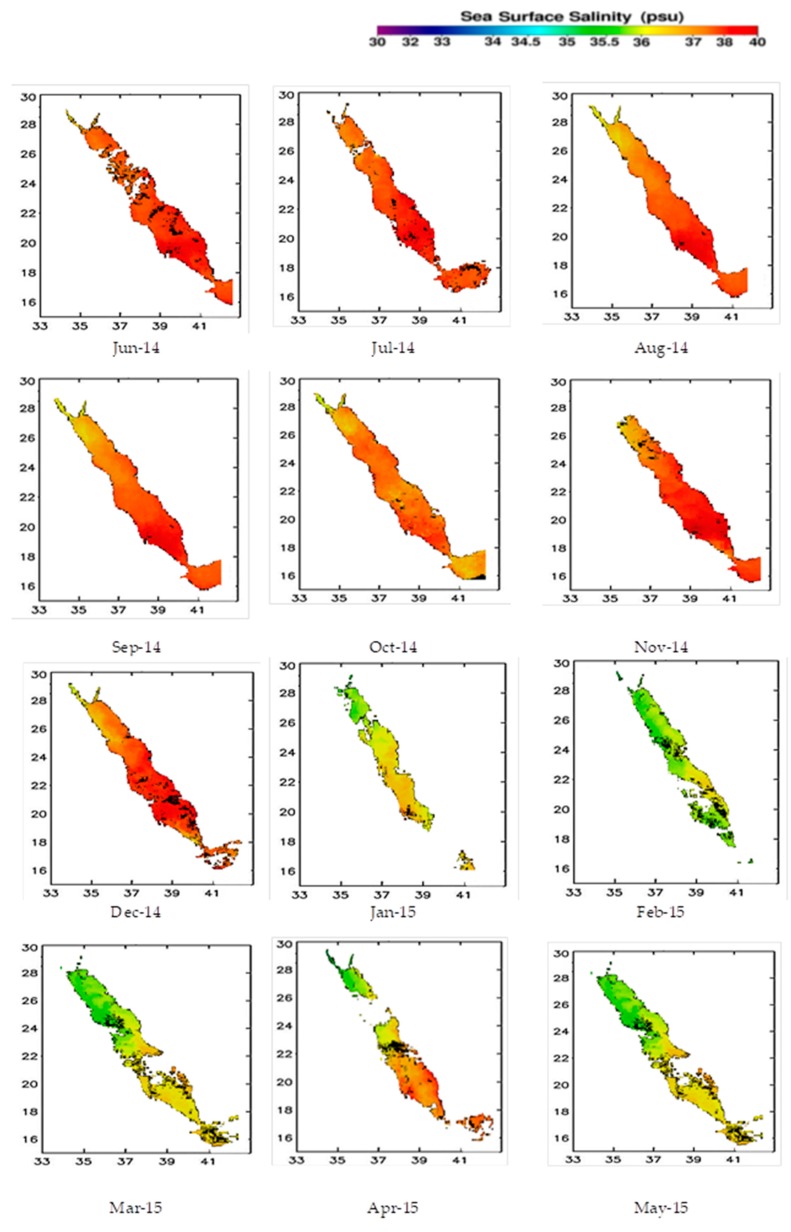
Average SSS distribution from MODIS-Aqua data (June 2014–May 2015).

The SSS multiple linear regression model developed in this study was subjected to validation with in situ measurements. To this end, the regression modeling approach was employed. The model has shown promising results as indicated by the corresponding scatter plots (Figure 3). The MODIS SSS estimates and in-situ SSS values were compared through numerous analyses, which revealed a strong correlation (r^2^ = 0.913, *p* < 0.01) and a 1.7 root mean square error (RMSE) found between the two datasets. It is worth noting that lower values were found in the MODIS-based SSS data compared to the in-situ data. The results of the validation test for the two datasets are presented in Figure 3, with the MODIS images and in-situ SSS values being identical in terms of the salinity class. The MODIS-based SSS distribution maps for the selected study area from June 2014 to May 2015 are illustrated in Figure 4. These months represent the monsoon and inter-monsoon periods along the Saudi Red Sea coast. The graph indicates that the south coast featured higher SSS values than the north coast, with this difference being more prominent between August and September 2013. The northern end of the Red Sea is located in rain shadow, meaning that the parameters of interest are not significantly impacted by the change in climate over the summer months. The coast of the Arab Sea, in the south, is less turbulent than the northern end of the Red Sea coastline [41], which explains the lower SSS values in the north, with this disparity being even more pronounced offshore. Higher SSS concentrations are also seen on the south coast compared to the east coast [42]. When the depth was taken with the vertical distribution average near shore (1–2 m), and open sea (20–100 m), the water depth variation of the sea bed depth at the near-shore and open sea affected the reflectance ranges in the two cases.

**Figure 5:**
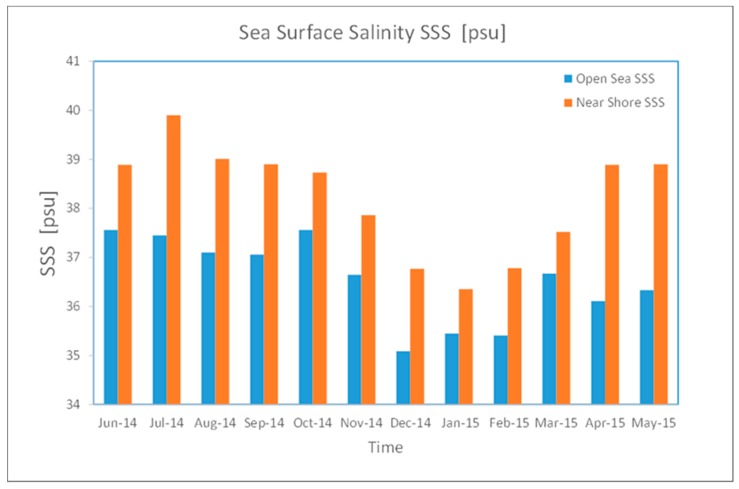
Average monthly SSS values for open sea and coastal waters using MODIS data (June 2014–May 2015).

According to Figure 5, SSS values of the near-shore have higher values during the summer months, with SSSmax values of 35.89 for open-sea samples and 36.17 for coastal samples in the winter, compared to the highest SSSmax scores of 38.87 for open sea samples and 39.98 for coastal samples during the summer. Large bodies of fresh water from coastal precipitation resulted in higher SSS in the open sea compared to the coastline over the winter months. In February 2014, the SSS value for the open sea water was 34.89, rising to 39.8 in August. Along the coastline, the SSS value was 36.25 in January 2014, rising to 38.32 in July. In May 2014 (inter-monsoon season), SSS values were 36.51 in the open sea and 35.45 near the coastline. Table 2 outlines the results of the analysis of 10 MODIS scenes from the northern and southern ends of the selected Red Sea study zone, with SSS estimates. The range of the near-shore is about 500 to 5000 m, and more than 10,000 is the open sea.

The northern end of the research site had an overall average SSS value of 34.63, with minimum values of between 33.01 and 36.25. The average SSS value increased to 35.48, with minimum values of between 35.17 and 35.98 during the inter-monsoon season. There was no significant difference between the minimum SSS values of late winter (March 2014) to the start of the inter-monsoon season (April 2014). Similar findings were obtained with regards to the maximum SSS values in the north, with the difference between the values being minimal. In the south, the average SSS value was 37.36, with minimum values between 34.82 and 39.90 in the late winter. The average SSS value increased to 38.41, with minimum values of between 37.1 and 39.53 during the inter-monsoon season. The average monthly SSS values for samples from both the open sea and coastal waters are presented in Figure 5, based on MODIS data from June 2014 to May 2015. The overall findings of the SSS results show that the difference in SSS values between near-shore SSS when compared to open-sea SSS range from 0.58 to 1.34 for minimum SSS values, and from 2.13 to 3.07 for maximum SSS values. Although the difference is small, all the measurements show higher SSS values for near-shore SSS when compared to open-sea SSS.

It is asserted in the literature that it is important to develop models that take environmental changes, such as seasonality, into account in order for the model to be used in various different regions around the world [26,33], Salleh et al.’s [24] model was developed using a small sample across a single season, with the model being relatively location-specific based on the results of the current study. Additionally, Salleh et al.’s model did not incorporate in-situ data from the monsoon season. Conversely, the model developed in the current study takes seasonality into account and uses a larger sample (total of 96 stations) taken from a larger area. After testing the model, a strong correlation (r^2^ = 0.913, *p* < 0.01) was found between the in-situ data and the values generated by the model.

### 4.2. SST: In-Situ versus MODIS

**Figure 6:**
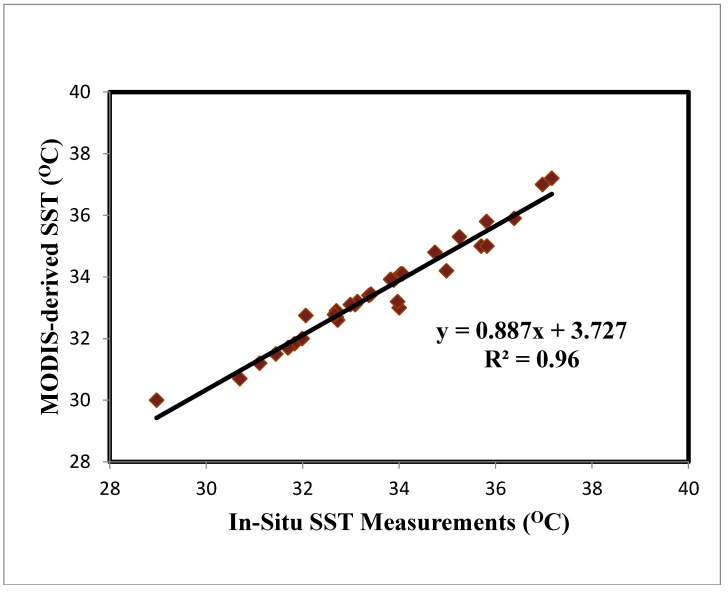
In-situ versus MODIS-based SST (°C) value.

The MODIS SST data for the selected Red Sea study site was compared to the in-situ data extracted during field visits conducted during the satellite passing period, with a strong correlation (r^2^ = 0.96, *p* < 0.01) and RMSE value of 0.154 °C between the two datasets. Overall, the SST values obtained from the in-situ samples were higher than the MODIS-based values. The average monthly coefficients between the chosen parameters and the SST are outlined in Figure 6.

**Figure 7:**
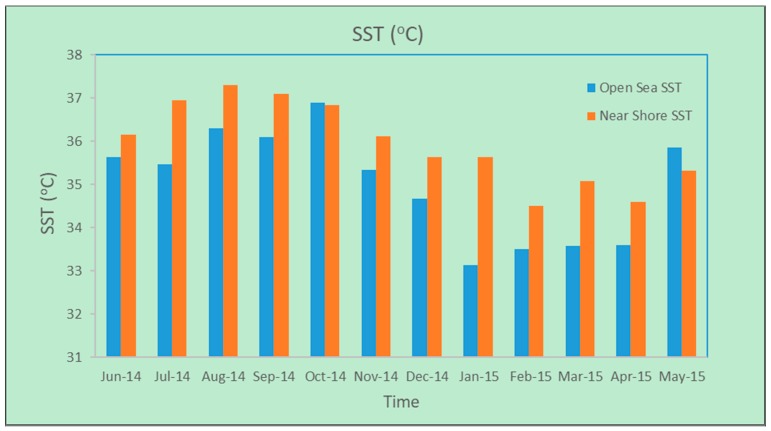
Average monthly SST (°C) measurements for open sea and coastal waters using MODIS data (June 2014–May 2015).

Average monthly SST (°C) values from the open-sea and coastal waters based on MODIS data from the selected study period are illustrated in Figure 7. The difference between mean SST values for a coastal reef zone of 96 km^2^ and a similar area 10 km offshore was used to calculate the cross-shore temperature gradient at Thuwal village, north of Jeddah. MODIS Aqua and Terra data were averaged into 5-d bins, but it was not possible to estimate the SST gradient for some of the intervals due to missing data. The cross-shore temperature gradient at Rayyis, south of Yanbu’ al Bahr, was calculated based on onshore and offshore zones of 64 km^2^ each, situated 10 km apart. The minimum and maximum SST values for the selected sites during the chosen periods are outlined in Table 3.

**Figure 8:**
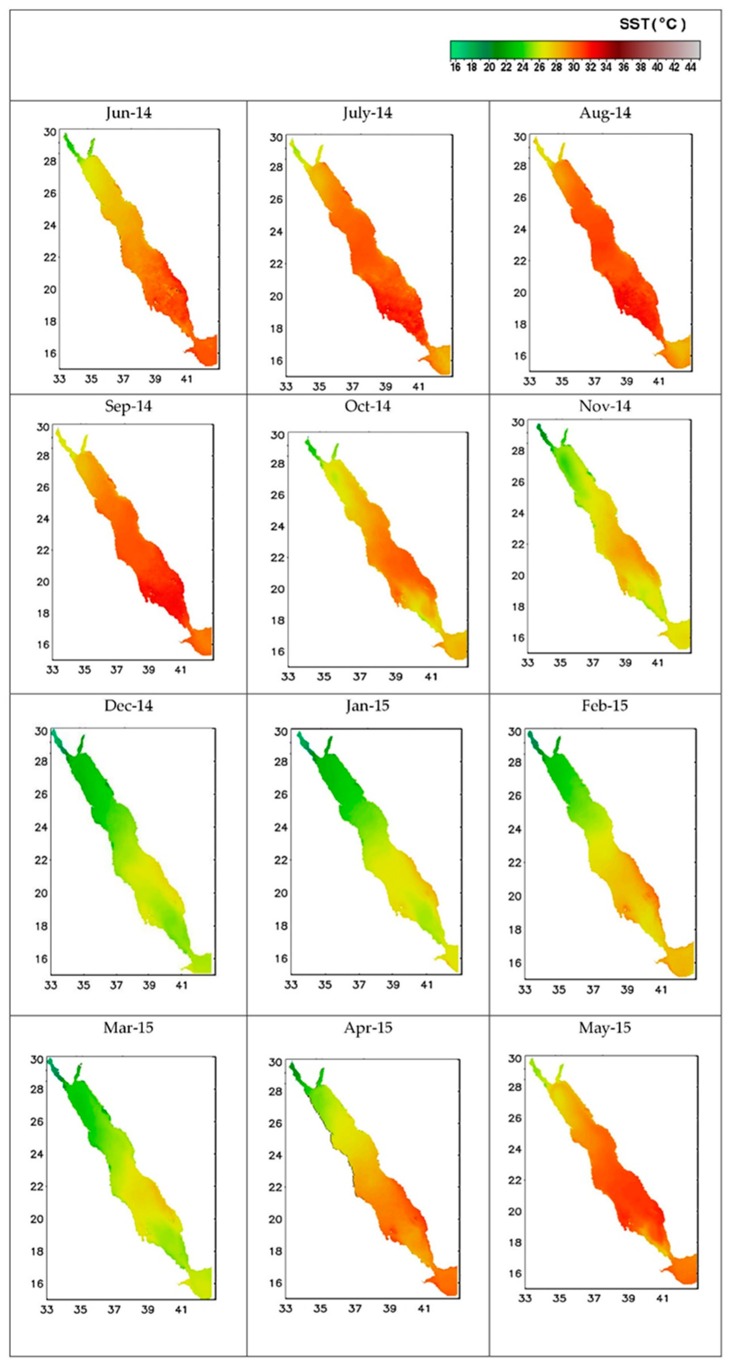
Average SST distribution based on MODIS-Aqua data (June 2014–May 2015).

The average monthly SST values from June 2014 to May 2015 for the selected Red Sea study sites are shown in Figure 8, obtained using MODIS data and presented using ERDAS and Carder’s algorithm [20]. The finding is in line with [26] and the recommendation that Carder’s model is the most suitable when comparing in-situ and MODIS-based data. The northern Red Sea zone demonstrated significant seasonal changes based on MODIS L3 SST. The SST ranged from 22 °C (most prominent in the northwest during March) and 32 °C (most prominent in the southeast during August). These findings are supported in the work of [43], who noted the impact of the surface wind regime moving from the northwest to the southeast; as well as [44], who described the atmospheric and meteorological factors that regulate Red Sea phytoplankton growth; and [25], who described surface thermohaline cyclonic circulation in the area; and [45,46], who noted total atmospheric heat loss.

Seasonal differences were found to be around 2 to 3 °C greater around the Gulf of Suez (particularly in shallow waters) compared to other areas of the northern Red Sea, with the Gulf of Aqaba and other deeper water zones being 1 °C lower. Winter months also brought a greater impact on SST from wind stress along the shoreline in comparison to other months. Conversely, SST was more significantly impacted by open water surface solar radiation during the spring and autumn than in the summer and winter months, with this being due to the dryness of the region. Latent heat loss can increase as a result of coastal breeze in the area, with evaporation increasing as air is forced over the surface by strong offshore winds. This finding is corroborated by [47], who stated that heat loss is significantly impacted by strong coastal winds during the study period at a buoy located 60 km away from the study site.

### 4.3. Chl-a: In-Situ versus MODIS

**Figure 9:**
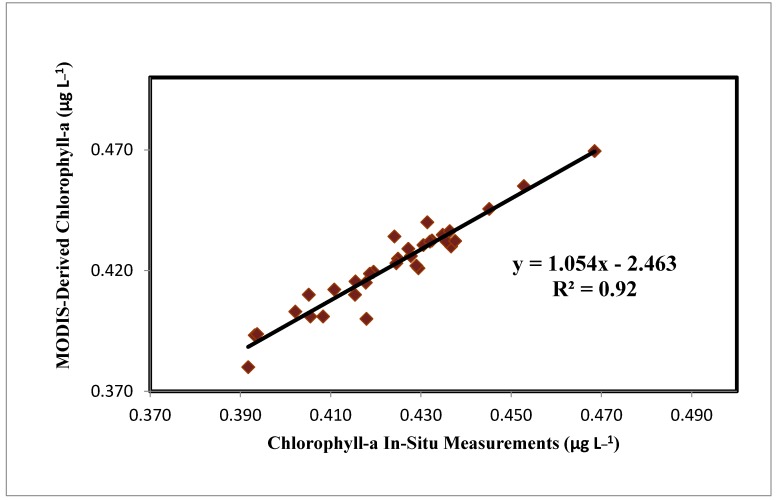
In-situ versus MODIS-based Chl-a concentration based on Carder’s algorithm (r^2^ = 0.92).

Figure 9 illustrates the comparison of Chl-a concentrations based on MODIS data and in-situ measurements from June 2014 to May 2015, using Carder’s algorithm [20]. Average monthly in-situ values ranged from 0.37 µg L^−1^ to 0.48 µg L^−1^, in line with [48]’s value range of 0.1 to 1.0 µg L^−1^. Validation of the model was conducted during the satellite pass. A strong positive correlation (r ≅ 0.93, *p* < 0.01) was found between the in-situ Chl-a values and the estimates from Carder’s algorithm [20], with an RMSE of 0.167 µg L^−1^, demonstrating the suitability of the model for Red Sea Chl-a measurement at the sea surface.

**Figure 10:**
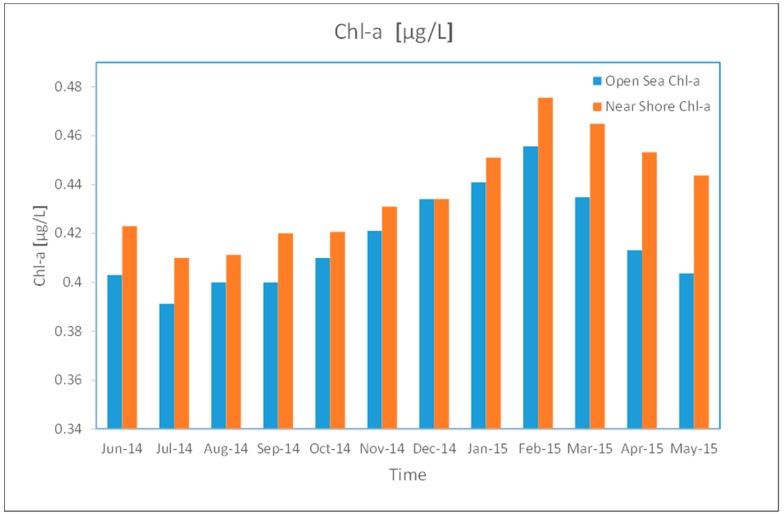
Average monthly Chl-a values (mg/L) for open sea and coastal waters using MODIS data (June 2014–May 2015).

Monthly average Chl-a values for coastal and open sea waters are illustrated in Figure 10, showing a range of 0.40 to 0.48 µg L^−1^ during the cooler months (January and February) and 0.37 to 0.42 µg L^−1^ during the warmer months (July and August), indicating higher Chl-a concentrations during the latter period. This is likely due to coastal waters being calmer during the summer season than in the winter. Additionally, reflectance from the grass is higher along the coast during the warmer months due to shallow shore water. These points are in line with the findings of [49] as well as the findings presented in the current paper regarding the meridional SST fronts.

Open sea Chl-a concentration was found to remain relatively stable at the sea surface between seasons, likely due to calmer waters in the nearshore year-round. For instance, during the warmer months, Chl-a was at an average concentration of 0.40 µg/L-1, ranging from 0.38 to 0.40 µg L^−1^, whilst during the colder months, Chl-a concentration was at an average of 0.43 µg L^−1^, with a range of 0.39 to 0.46 µg L^−1^. Strong vertical mixing occurs in the open sea during the winter months (November–March) as a result of strong wind forces and cold weather, resulting in an increase in Chl-a levels in this part of the water over this period. During April (inter-monsoon season), the growth of zooplankton then follows in the open sea. In shallow waters, colder weather results in Chl-a growth being largely suspended until the summer months begin. As a result of heavy precipitation between November and February, high cloud cover can be seen in the Chl-a map for the selected study site over the winter months, making MODIS data extraction challenging when using only one or two bands.

**Figure 11:**
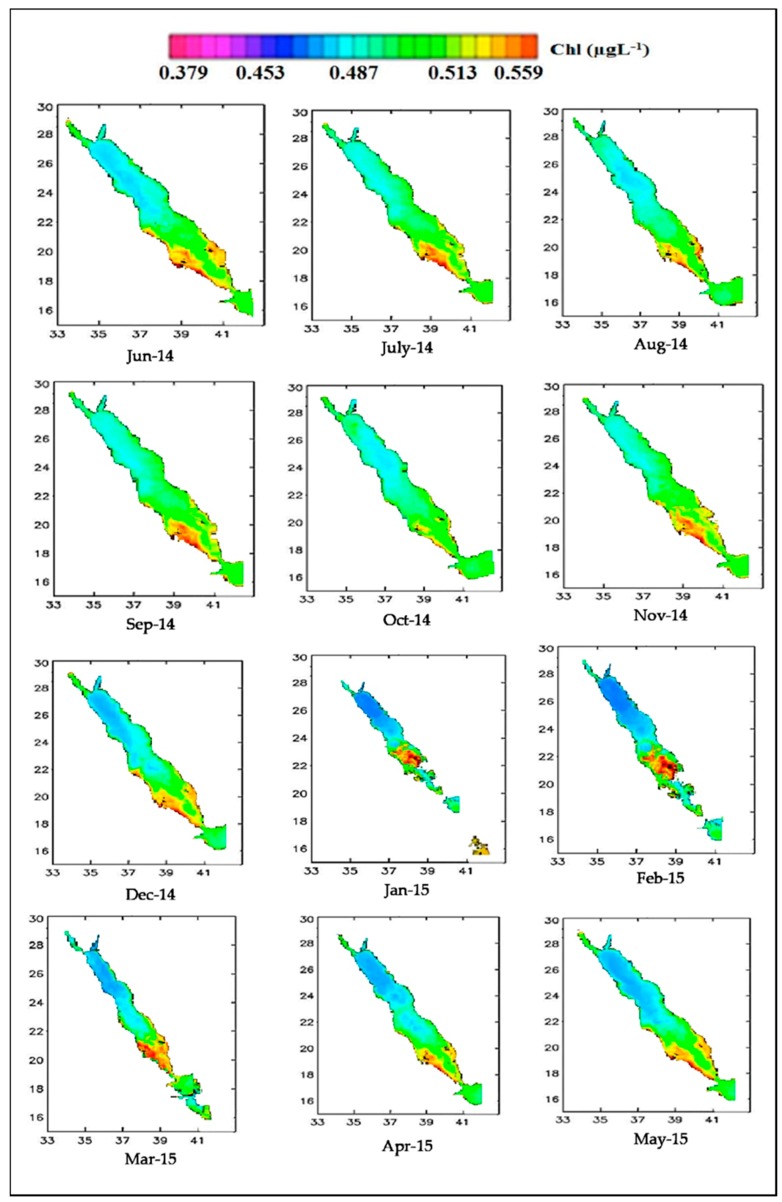
Average Chl-a distribution based on MODIS-Aqua data (June 2014–May 2015).

ERDAS software was used to generate maps using Carders’s algorithm [20] on MODIS Chl-a concentration data for the focus period of June 2014 to March 2015, as shown in Figure 11. As noted earlier, Carder’s algorithm was selected based on the recommendation of [26], who asserted that the model is most suitable for analyses dealing with in-situ and MODIS-based Chl-a data. Seasonal differences in Chl-a concentrations were found in the northern Red Sea, with the highest (>0.49 µg L^−1^) found during the winter season and particularly along the eastern shoreline. As in the previous case, the findings are in line with the earlier work of [44,50] regarding meridional SST fronts.

The results indicate that the southern and northern zones of the Red Sea experience slightly elevated Chl-a concentrations during the winter season compared to the summer season, with colder months associated with higher Chl-a values and warmer months associated with lower Chl-a values based on the MODIS L3 Chl-a data. Data for the summer season shows average Chl-a concentration levels of 39.65 µg L^−1^, with a range of 0.37 to 0.40 µg L^−1^. In the nearshore, seasonality appeared to have no significant impact on Chl-a levels at the sea surface. This is likely due the stable nature of the waters year-round in this area of the Red Sea. The finding is corroborated by Acker et al. [50], who indicated that Chl-a concentration was higher in the northern end of the Red Sea than the southern end during the period of study. Thus, it can be stated that the productivity is greater in the northern Red Sea during the colder winter months, with Chl-a concentration levels of 0.33 to 0.70 µg L^−1^, as stated in the early research of [51], and the more recent study conducted by [44,52,53]. Seasonal differences also appear to have an impact around the Gulf of Aqaba to the northern end of the Red Sea, with the hottest months associated with a low Chl-a of under 0.14 µg L^−1^. Consequently, the results of this study demonstrate that Chl-a levels appear to be higher in the colder winter period and lower in the hotter summer period around the northern near-shore waters. It is worthy to note that the Chl-a levels in winter are high only in a limited part of the sea (Figure 11), which might raise the average values (Figure 10).

### 4.4. PFZ Determination Based on SSS, SST, and Chl-a

**Figure 12:**
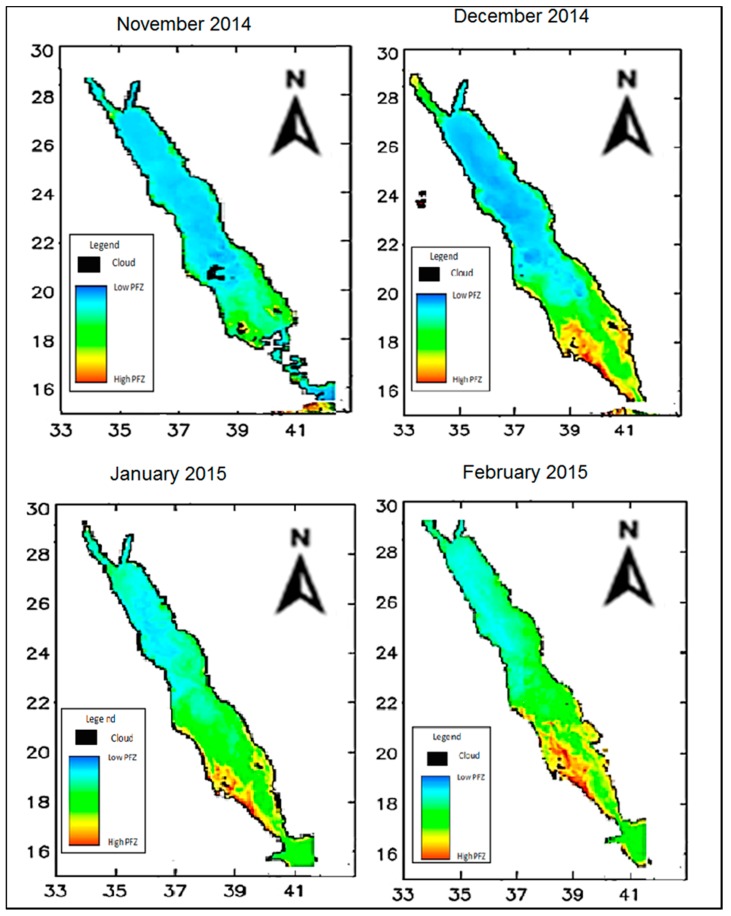
PFZ mapping across the Red Sea study site from early winter to the end of the monsoon season (November 2014–February 2015).

The main aim of this research was to determine the usefulness of SSS, SST, and Chl-a measurements in determining PFZs across the Red Sea. Chlorophyll is recognized as the most important oceanographic factor impacting the marine environment and the natural habitat of fish species. In the adopted model, Chl-a and SST were closely weighted as they are taken to share a similar level of influence over the mapping of potential fishing zones. MODIS-based SST data were found to be highly accurate in relation to Chl-a values. However, fish habitat continues to be defined by measures of nutrients in the water, sea temperature, and water salinity. Zones considered to have high potential as fishing areas are assumed to have a large population of available fish stock due to aggregation. Figure 12 illustrates PFZ mapping across the selected Red Sea study site between November 2014 (the beginning of winter season) and February 2015 (the end of monsoon season).

As noted earlier in this paper, Chl-a levels begin to increase between November and March near the southern end of the Red Sea and in the open waters, with the inter-monsoon month of April bringing zooplankton growth. This occurs as a result of strong vertical mixing due to high wind forces and cold weather during the winter months, thereby impacting fish aggregation and influencing the amount of available fish stock for fisheries, with high fish aggregation levels found in the open waters of the Red Sea during the winter season. The results of the current study demonstrate that in the northern Red Sea region, both SSS and SST decrease during the winter as a result of freshwater brought in through precipitation over these months. The model adopted in this research, therefore, suggests that the chosen oceanographic parameters (SSS, SST, and Chl-a) influence fish aggregation during the summer and winter seasons. This finding could be of significant value for fisheries in Saudi Arabia and other countries bordering the Red Sea.

The FPZ mapping images are shown in Figure 12, which indicate that the open waters of the Red Sea are prime potential fishing areas during the late summer and early winter months. Fish populations migrate south towards the coastal waters of the Arabian Gulf, where water temperatures are higher. The effect of high SST, SSS, and Chl-a concentration on fish migration is more noticeable in the southern and open waters, and as a result, a higher level of fish aggregation is found in these parts in comparison to fish aggregation levels in northern waters and areas closer to the shoreline. The bottom topography along with the regulating atmospheric and meteorological parameters control the Red Sea fish aggregation. These factors comprise of the horizontal transfer of upwelling water through eddy circulation and possible mineral fertilization from atmospheric dust deposition. According to [25], a combination of conditioning factors contributes to anomalous phytoplankton events in the Red Sea basin and might have affected various oceanographic attributes, including sea surface chlorophyll, temperature, and salinity. The higher temperature that leads to an increasing evaporation rate of seawater is associated with the higher salinity values observed in summer months, and both the near-surface temperature and salinity may experience seasonal fluctuations associated with the impact of monsoon winds on the circulation of the Red Sea. Strong south-southeasterly winds prevail over the southern Red Sea in winter months and result in a surface mass flux of relatively colder fresh water, which could bring nutrients, leading to enhanced phytoplankton that normally blooms during winter. On the contrary, for the period of summer months, north-northwesterly winds tend to extend over the Red Sea and may drive more saline surface water in the northern Red Sea to the south [25,41,43,47].

These points are reflected in the results of the current study based on the PFZ maps for the winter months, highlighting the influence of Chl-a, SST, and SSS on fish aggregation. Specifically, the increase in Chl-a levels in the water over the colder months is shown to result in higher fish aggregation based on the movement of PFZs. As illustrated in the PFZ maps above, during the warm summer months when low rainfall leads to a decline in Chl-a, an increase in SST, and decrease in SSS, a decrease in PFZs occurs. This is because greater salinity and warmer temperatures coupled with lower chlorophyll make the environment inhabitable for fish. The PFZ model featured a positive relation with Ch1-a, and a negative correlation relationship with both SST and SSS. It is evident that anomalously cold, nutrient-rich water that either “wells up” from below or surface mass influx from colder regions due to the impact of strong south-southeasterly winds that prevail over the southern Red Sea in winter seasons, exhibit relatively colder fresh water, which could enhance phytoplankton blooming. On the other hand, anomalously warmer water during summer months causes a decrease in phytoplankton blooming due to stratification, reduction in sea layer mixing, and eventually results in nutrient shortages. The impact of the monsoon season on fish aggregation is also demonstrated by the PFZ maps, illustrating the influence of SSS, SST, and Chl-a on fish aggregation and the availability of PFZs. Here, it can be seen that the winter season is associated with a higher level of PFZs compared to other months of the year, with the beginning of the monsoon season being one of the most beneficial periods for fishing. Salinity levels are significantly higher in shallower coastal waters than they are in deeper open waters during the summer months, with this difference being less prominent during the winter. During the monsoon season, better water temperatures, salinity and depth result in more substantial PFZs away from the shoreline compared to waters along the coast. Additionally, the high-aggregation open waters of the Red Sea were found to have higher SST and Chl-a values compared to the more northern areas of the Red Sea.

### 4.5. Validation Testing

**Figure 13:**
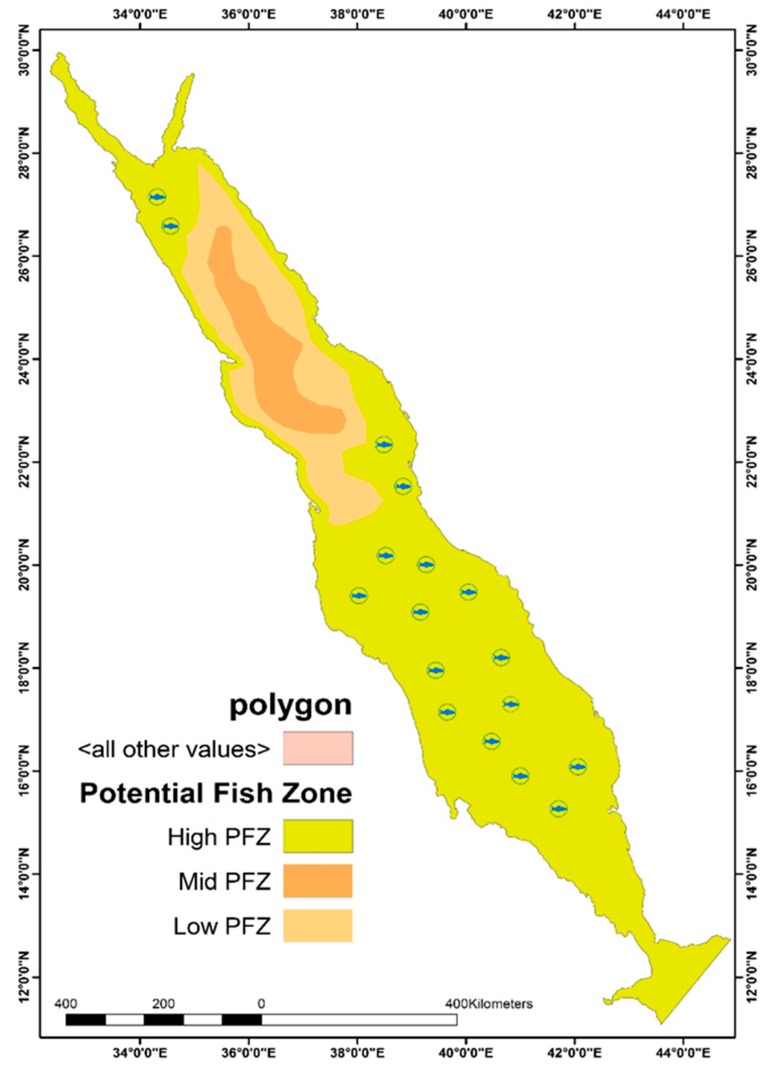
PFZ map of the Red Sea generated by the proposed model (July 2014).

The PFZ model presented in this paper was validated with the MODIS-based PFZ, along with the in-situ GIS-based data (Figure 13), where fish symbols represent the fish aggregation locations acquired from Saudi Ministry of Environment, Water, and aAgriculture, with good reliability found based on the results of the validation test. Given this, the model developed in the current study could be of value for Saudi fisheries working around the Red Sea coastline. Figure 13 presents a PFZ map generated by the proposed PFZ model for the month of July 2014, illustrating high PFZ areas that match the PFZs generated by the MODIS-based model. In line with the discussion in the previous section, open waters are shown to have higher potential as fishing zones compared to waters along the coastline.

The above PFZ map was generated using SST, SSS, and Chl-a data collected in July 2014 and validated using fish landing data gathered from 50 stations on the same day that the map was generated. The fish landing and PFZ model data can be seen to have a high level of similarity, highlighting the potential accuracy and value of the proposed model. These results also highlight the significance of the three oceanographic parameters studied in this research (i.e., SSS, SST, and Chl-a) with regards to the determination of PFZs in the Red Sea. In line with the findings of Salleh et al. [26], it has been emphasized in the current paper that seasonal shifts in these parameters can result in environmental changes that impact fish aggregation, therefore impacting the availability of fish for fisheries during the summer and winter months.

## 5. Conclusions

This study was conducted in recognition of the increasing demand for fish as an essential food source, the pressure this has placed on local fisheries, and the need to utilize modern technologies to maximum impact in order to support the fishing industry and make the best use of ocean resources. Therefore, a novel linear regression model was developed as part of this project, using MODIS-based data to estimate SSS values in the selected Red Sea study site. This study also confirms the suitability of the Hu model for Chl-a estimation and the Walton model for SST estimation when dealing with MODIS-based data. The results of this study also demonstrate that a correlation exists between Chl-a and SSS levels. The analyses performed during the research also confirm the presence of seasonal impacts on fish aggregation, with the monsoon season being of particular importance in the selected Red Sea region. After performing validation testing on the proposed PFZ model, the results indicate that the model developed during this project could be of much value for fisheries around the Red Sea region, and potentially also for fisheries in other locations around the world. The predictive ability of the proposed model was found to be 95.5%, whilst the previous model was rated with a 56% predictive ability.

## Figures and Tables

**Table 1:** Quantitative fish catch data for Red Sea.

Station	Red sea Region	Latitude N	Longitude E	Fish Catch kg in July 2014	Fish Catch per year 2014/ton	Fish Catch per year 2015/ton	Fish Catch Quality
1	North	27.22°	35.26°	80,520	711	677	Mid-PFZ
2		27.06°	35.21°	91,541	813	455	Mid-PFZ
3		26.58°	35.28°	76,589	576	387	Low-PFZ
4		26.48°	35.32°	49,209	210	280	Low-PFZ
5		26.65°	35.70°	21,342	188	140	Low-PFZ
6		26.32°	35.62°	28,765	210	110	Low-PFZ
7		26.08°	36.09°	57,291	389	645	Mid-PFZ
8		24.99°	36.48°	36,178	310	189	Low-PFZ
9		23.77°	37.98°	62,434	430	140	Low-PFZ
10		21.70°	38.22°	47,623	390	130	Low-PFZ
Total				551,492	4227	3153	
11	Central	20.90°	38.74°	84,218	812	895	High-PFZ
12		20.88°	38.99°	34,176	635	548	Mid-PFZ
13		20.67°	39.06°	13,178	376	495	Mid-PFZ
14		20.42°	39.04°	84,954	451	512	Mid-PFZ
15		20.49°	39.31°	63,498	289	863	High-PFZ
16		20.38°	39.23°	74,673	567	378	Low-PFZ
17		20.35°	39.22°	48,629	732	678	Mid-PFZ
18		20.33°	39.21°	73,621	298	180	Low-PFZ
19		20.23°	39.17°	83,951	387	469	Mid-PFZ
20		20.31°	39.20°	52,163	721	737	High-PFZ
Total				613,061	5268	5755	
21	South	38.83°	40.04°	85,398	922	735	High-PFZ
22		18.22°	40.80°	13,278	431	956	High-PFZ
23		18.26°	41.21°	89,532	940	739	Mid-PFZ
24		18.54°	40.68°	62,398	732	493	Mid-PFZ
25		17.81°	40.82°	231,788	3541	1891	High-PFZ
26		17.37°	41.56°	95,436	632	250	Low-PFZ
27		17.48°	40.89°	84,329	432	759	High-PFZ
28		16.95°	42.14°	71,474	563	689	Mid-PFZ
29		16.93°	41.48°	257,912	2890	1890	High-PFZ
30		16.65°	41.58°	27,969	843	915	High-PFZ
Total				1,019,514	11926	9317	
100–390: Low-PFZ							
390–750: Mid-PFZ							
750 and up: High-PFZ							
Source: Ministry of Environment, Water and Agriculture							

**Table 2:** Minimum/maximum Red Sea SSS Values (June 2014–May 2015).

Date of Satellite Image	Near-Shore SSS ()		Open Sea SSS ()	
	Min	Max	Min	Max
June 14	38.14	38.89	36.76	37.56
July 14	38.98	39.90	36.88	37.45
August 14	38.25	39.01	36.65	37.10
September 14	37.78	38.90	36.10	37.06
October 14	37.34	38.73	36.02	37.56
November 14	36.89	37.86	35.06	36.65
December14	36.24	36.77	34.56	35.09
January 2015	35.66	36.35	34.69	35.45
February 2015	35.12	36.78	35.14	35.41
March 2015	35.56	37.52	34.95	36.67
April 2015	37.45	38.89	35.82	36.11
May 2015	37.23	38.90	35.95	36.33

**Table 3:** Minimum and maximum SST (°C) values for open sea and coastal waters (March 2014–February 2015).

Date of Satellite Image	Near Shore SST (°C)		Open Sea SST (°C)	
	Min.	Max.	Min.	Max.
March 2014	33.20	33.97	34.97	35.20
April 2014	33.10	34.09	34.09	35.10
May 2014	35.80	35.90	35.02	35.60
June 2014	35.40	35.88	35.90	36.40
July 2014	35.22	35.70	36.20	37.70
August 2014	35.71	36.88	36.71	37.90
September 2014	35.20	36.98	36.98	37.20
October 2014	36.83	36.96	36.83	36.86
November 2014	35.00	35.66	36.00	36.23
December 2014	34.60	34.73	35.55	35.70
January 2015	32.90	33.38	35.38	35.90
February 2015	33.00	34.00	34.00	35.00
